# Immunohistochemical Analysis of CYP11B2, CYP11B1 and β-catenin Helps Subtyping and Relates With Clinical Characteristics of Unilateral Primary Aldosteronism

**DOI:** 10.3389/fmolb.2021.751770

**Published:** 2021-09-24

**Authors:** Luyan Sun, Yiran Jiang, Jing Xie, Hongyuan Zhu, Luming Wu, Xu Zhong, Weiwei Zhou, Tingwei Su, Weiqing Wang

**Affiliations:** ^1^ Shanghai Key Laboratory for Endocrine Tumors, Shanghai Clinical Centre for Endocrine and Metabolic Diseases, Shanghai Institute of Endocrine and Metabolic Diseases, Ruijin Hospital, Shanghai Jiaotong University School of Medicine, Shanghai, China; ^2^ Department of Pathology, Ruijin Hospital, Shanghai Jiaotong University School of Medicine, Shanghai, China; ^3^ Laboratory for Endocrine and Metabolic Diseases of Institute of Health Science, Shanghai Jiaotong University School of Medicine and Shanghai Institutes for Biological Sciences, Chinese Academy of Sciences, Shanghai, China

**Keywords:** hyperaldosteronism, CYP11B2, CYP11B1, beta catenin, immunohistochemistry, prognosis

## Abstract

**Background:** Primary aldosteronism is caused by aldosterone overproduction. While conventional hematoxylin-eosin staining can demonstrate morphological abnormality, it cannot provide any functional histopathological information. We aimed to identify the diagnostic, functional and prognostic value of CYP11B2, CYP11B1, and β-catenin immunostaining in unilateral hyperaldosteronism.

**Method:** A total of 134 patients with unilateral hyperaldosteronism were recruited in our study. The expression of CYP11B2, CYP11B1, and β-catenin was evaluated semiquantitatively on 134 patients’ sections using immunohistochemistry technology and the relationship with clinical data was assessed.

**Results:** Patients were classified into four subtypes based on CYP11B2 staining as below: (1)118 patients with unilateral single aldosterone-producing adenoma (APA), (2)11 with unilateral multiple APA, (3)four with aldosterone-producing cell cluster (APCC), and (4)one with an undefined source. Adjusted CYP11B2 H-score was correlated with serum aldosterone, aldosterone to renin ratio (ARR), and serum potassium. In the abnormal β-catenin staining group, hypertension duration, aldosterone, ARR, cortisol, tumor diameter, tumor area, and CYP11B2 H-score were significantly higher than those of the wild-type group. Serum potassium level was significantly lower in the abnormal β-catenin staining group. Age, gender, BMI, family history of hypertension, adjusted CYP11B2 and CYP11B1 H-scores differed significantly between complete clinical success and incomplete clinical success groups. Age, gender and family history of hypertension were independently associated with complete clinical success based on multivariate logistic regression analysis.

**Conclusion:** CYP11B2 immunostaining could improve the differential diagnosis of unilateral hyperaldosteronism. Adjusted CYP11B2 H-score could be used as a histopathological marker to reflect the severity of unilateral APA. Dysregulation of Wnt/β-catenin signaling and impaired β-catenin degradation may provoke the proliferation and enhance the steroidogenic ability of APA tumor cells, indicating that the Wnt pathway might be a potential, actionable, therapeutic target in the treatment of hyperaldosteronism. Age, sex and family history of hypertension were independent predictors of clinical outcome after adrenalectomy for unilateral hyperaldosteronism.

## Introduction

Primary aldosteronism (PA) is a group of disorders caused by an autonomous overproduction of aldosterone from the adrenal glands, independent of the renin-angiotensin system (Stowasser and Gordon, 2016). PA is the most common form of secondary hypertension, with an estimated prevalence reaching more than 10% in the hypertensive population (Rossi et al., 2006) and even approximately 20% in resistant hypertension patients (Calhoun et al., 2002).

Conventional hematoxylin-eosin (HE) staining performed in most pathological laboratories provides only morphological information; however, it is unsuitable for functional histopathological analyses (Nakamura et al., 2014). For instance, the differential distinction between an adenoma and a nodular hyperplasia is unclear on HE staining because many aldosterone-producing adenomas (APAs) also exhibit peritumoral zona glomerulosa (ZG) hyperplasia (Ganguly, 1998; Enberg et al., 2004; Boulkroun et al., 2010; Yamazaki et al., 2017). CYP11B2 and CYP11B1, which catalyze the final biosynthesis steps of aldosterone and cortisol, respectively, share a 93% homogeneity at the amino acid sequence level (Gomez-Sanchez et al., 2014). With the successful development of specific antibodies against CYP11B2 and CYP11B1 (Gomez-Sanchez et al., 2014), immunohistochemistry is now a promising technique for precisely locating the culprit responsible for aldosterone excess and for broadening our knowledge regarding the spectrum of PA. Nishimoto et al. (Nishimoto et al., 2010) have revealed an entity of subcapsular CYP11B2-expressing cells, termed aldosterone-producing cell cluster (APCC), which are 200–1,300 μm wide and 100–500 μm deep and can sustain aldosterone secretion in spite of a suppressed renin-angiotensin-aldosterone system. Later, Omata et al. (Omata et al., 2018) demonstrated that bilateral idiopathic hyperaldosteronism may result from the accumulation and enlargement of APCCs which harbor somatic aldosterone-driver gene mutations rather than diffuse aldosterone-producing ZG hyperplasia. Hence, CYP11B2 immunostaining can markedly improve the accuracy of PA histopathological diagnosis.

The Wnt/β-catenin signaling pathway is critical for regulating the proliferation, differentiation, and tumorigenesis of the adrenal cortex (El Wakil and Lalli, 2011; Clevers and Nusse, 2012). And several studies have reported mutaion burden of CTNNB1 gene which encodes β-catenin in APA (Scholl et al., 2015; Akerstrom et al., 2016). The frequency of somatic CTNNB1 mutation in APA ranges from 2.1 to 5.1% (Scholl et al., 2015; Akerstrom et al., 2016). Most CTNNB1 mutations affect the serine/threonine residues in exon 3, which thereby hamper the proteasomal degradation of β-catenin under resting conditions, leading to abnormal intracellular accumulation of β-catenin. Actually, constitutive activation of Wnt/β-catenin signaling is associated with aldosterone overproduction and treatment of H295R cell line, which already has an activating mutation of CTNNB1 endogenously, with siRNA against β-catenin or β-catenin inhibitor can significantly decrease aldosterone secretion (Berthon et al., 2014). However, the mechanism underlying how β-catenin provokes mineralocorticoid overproduction is undetermined; possible explanations include the indirect activation of CYP11B2 promoters (Berthon et al., 2014) or increased expression of LHCGR and GNRHR by β-catenin in pregnant and postmenopausal patients (Teo et al., 2015).

Laparoscopic adrenalectomy is the first-line choice for treating unilateral PA and excess aldosterone secretion can be resolved in most cases (Stowasser and Gordon, 2016). But not all PA patients can achieve hypertension cure or remission after the surgery, the factors that may affect clinical outcome include age, gender, family history of essential hypertension, hypertension duration, BMI, antihypertensive drug dosage and so on (Steichen et al., 2012; Williams et al., 2017). Nevertheless, individual prediction of the clinical outcome based on the above factors is not completely accurate, and more predictors based on new emerging technology need to be identified (Stowasser and Gordon, 2016). To date, the association between immunostaining features and prognosis of unilateral PA after surgery still remains an uncharted area and warrants further investigation.

Therefore, the aim of our current study is to utilize CYP11B2 immunostaining to subtype clinically diagnosed unilateral PA and explore the functional and prognostic significance of CYP11B2, CYP11B1 and β-catenin immunostaining.

## Patients and Methods

### Patients

We recruited 134 patients referred to the Shanghai Clinical Centre for Endocrine and Metabolic Diseases in Ruijin Hospital Affiliated to Shanghai Jiaotong University School of Medicine from February 2010 to January 2013. The study protocol was approved by the local ethics committee and informed consent was obtained from all patients.

All patients underwent a diagnostic workup that included measurement of serum ions (Na^+^, K^+^, Cl^−^, P, and Ca^2+^), serum cortisol, plasma renin activity (PRA), and serum aldosterone. Diagnostic workup of PA was confirmed according to the 2008 Endocrine Society Clinical Practice Guideline as previously described (Funder et al., 2008; Jiang et al., 2015). First, patients were required to withdraw mineralocorticoid receptor antagonists and non-potassium sparing diuretics for at least 4 weeks. In addition, β-blockers, angiotensin-converting enzyme inhibitors, and angiotensin II receptor blockers were withdrawn for at least 2 weeks. Only non-dihydropyridine calcium blockers and/or α-receptor blockers were permitted to control blood pressure. Oral potassium supplementation was added if serum potassium level was less than 3.5 mmol/L. Second, aldosterone to renin ratio (ARR) was calculated for detecting suspicious cases of PA. Patients were kept in an upright posture for 2 h and then seated for 15 min before blood samples were collected in the morning. Third, a saline infusion test was performed in patients with ARR >30 (ng/dl)/(ng/mL h) as the confirmatory test for PA. Serum aldosterone was measured in a supine position before and after the infusion of 2L 0.9% NaCl solution from 8:00 am to 12:00 pm. Failure in suppressing post infusion aldosterone level to 10 ng/dl confirmed the diagnosis of hyperaldosteronism.

Adrenal computed tomography (CT) and adrenal venous sampling (AVS) were performed in patients with PA to differentiate between bilateral and unilateral PA. More details of AVS are described in the Supplementary Material. Patients with unilateral aldosterone overproduction underwent laparoscopic adrenalectomy.

Patients aging from 18 to 70 years old who underwent confirmation workup of unilateral PA (saline infusion test, CT scan and AVS) were included in our study. Patients who are concomitant with other endocrine diseases such as Cushing syndrome and pheochromocytoma, who have chronic renal failure, chronic heart failure, liver cirrhosis and have cardiovascular event attack within 6 months were excluded in our study. Also, pregnant patients were excluded. 156 patients were screened in our study. 22 patients were excluded according to exclusion criteria.

The diagnosis of adenoma and UAH were established according to the four corners criteria: 1) a biochemical confirmation of PA; 2) aldosterone secretion is lateralized, which is validated by AVS; 3)adenoma or nodular hyperplasia at histopathology; and crucially important, 4) with cure or improvement in blood pressure, aldosterone, plasma renin activity, ARR and serum potassium after adrenalectomy (Stowasser and Gordon, 2016). Pathological diagnosis was performed by two experienced pathologists. Primary Aldosteronism Surgical Outcome (PASO) criteria were used for outcomes and follow-up assessment of adrenalectomy for unilateral PA (Williams et al., 2017). Complete clinical success was defined as patients with normalized blood pressure without any use of antihypertensive medicine. Incomplete clinical success was defined as patients requiring antihypertensive medicine to control BP, with partial and absent clinical success defined according to PASO criteria. Blood pressure, serum ions (Na^+^, K^+^, Cl^−^, P, and Ca^2+^), aldosterone and plasma renin activity were measured as routine.

### Laboratory Assays

Laboratory assays were performed as previously described (Jiang et al., 2015). All tests were performed in a CAP (College of American Pathologists, No. 7217913)-accredited laboratory. Serum aldosterone and PRA were measured by radioimmunoassay following the manufacturer’s instructions (Beckman Coulter Corp., Brea, CA, United States). Plasma cortisol was measured on Access Immunoassay Systems (Beckman Coulter Corp., Brea, CA, United States).

### Immunohistochemistry

Immunohistochemistry was performed according to the standard histopathological protocol described in our previous study (Wu et al., 2018). Antibodies against CYP11B2 (mouse monoclonal, 1:200; Merck Millipore, MABS1251, RRID: AB_2650562), CYP11B1 (rat monoclonal, 1:200; Merck Millipore, MABS502, RRID: AB_2650563), and β-catenin (rabbit polyclonal, 1:400; Cell Signaling Technology, Cat #9562, RRID: AB_331,149) were used. A standard avidin-biotin-peroxidase complex technique was used to demonstrate the primary antibody binding (Vector pk-7200; Vector Laboratories, Inc., Burlingame, CA, United States). Here, an APA was defined as a CYP11B2-positive, well-circumscribed, round or ovoid-shaped nodular lesion composed of a mixture of ZG-like and zona fasciculata (ZF)-like cells, often with a fibrous capsule around it. In contrast, an APCC was defined as a CYP11B2-positive cell cluster, cuneiform or trapezoid in shape, and morphologically identical to adjacent ZG cells without a fibrous capsule. McCarty H-score was used to evaluate the immunoreactivity semi-quantitatively in our study, considering both the percentage of positively stained cells and intensity of their immunopositivity (McCarty et al., 1985; Nakamura et al., 2014; Ono et al., 2014; Monticone et al., 2015). More details of assessing McCarty H-score are described in the Supplementary Material.

As for β-catenin immunostaining, both the percentage of immunopositive cells and immunointensity of the stained cells were also recorded. Patients were categorized into the abnormal β-catenin staining group for focal cytoplasmic staining (<30% but strong staining), diffuse cytoplasmic staining (30–70% and at least distinct staining, >70% regardless of staining intensity), focal nuclear staining (<5% of nuclei but strong staining), or diffuse nuclear staining (>5% and at least distinct staining); otherwise, they were categorized into the wild-type staining group (Wu et al., 2018).

### Statistical Analysis

Continuous variables were expressed as median (interquartile intervals: 25–75%), while categorical variables were expressed as frequency and percentage. The Mann-Whitney *U* test was used for the comparison of continuous parameters between two different groups, and the chi-squared test was introduced for the comparison of categorical variables. Spearman’s rank correlation was employed to explore the association between clinical and immunostaining parameters. We performed univariate and multivariate logistic regression analyses to determine the predictors associated with complete clinical success. All statistical analyses were two-sided and performed using IBM SPSS Statistics (version 22.0) software. *p* < 0.05 was considered significant.

## Results

### Clinical and Hormonal Data of Patients With Unilateral PA

Overall 134 patients were enrolled in our study (Table 1). The median age at presentation was 48 years, with an approximately 78 months median duration of hypertension. The median systolic blood pressure (SBP) and diastolic blood pressure (DBP) were 165 and 100 mmHg, respectively. The median body mass index (BMI) was 23.19 kg/m^2^. Male patients accounted for 42.5% of all the subjects and 53.7% of the patients had a family history (first-degree relatives) of hypertension. The median tumor size on CT scan was 1.5 cm, and the median tumor area was 1.77 cm^2^. Additionally, the serum aldosterone concentration (median 44.75 ng/dl, range 28.32–68.27) and ARR (median 468.11, range 170.90–1929.33) were high and PRA was suppressed (median 0.10 ng/ml h, range 0.03–0.22). The median value of serum cortisol among the patients with PA was 11.49 μg/dl (range 9.08–14.92).

**TABLE 1:** Clinical characteristics of the study population.

	All patients (n = 134)
Age (years)	48 (40, 55)
Male (%)	42.5
Duration of hypertension (months)	78 (36, 120)
Family history of hypertension (%)	53.7
SBP (mmHg)	165 (150, 180)
DBP (mmHg)	100 (95, 110)
BMI (kg/m^2^)	23.19 (21.22, 25.51)
Aldosterone (ng/dl)	44.75 (28.32, 68.27)
Renin activity (ng/ml·h)	0.10 (0.03, 0.22)
ARR	468.11 (170.90, 1929.33)
Serum potassium (mmol/L)	2.47 (2.10, 2.86)
Prevalence of Hypokalemia (%)	97.0
Serum cortisol (µg/dl)	11.49 (9.08, 14.92)
Tumor diameter (cm)	1.50 (1.10, 2.00)
Tumor area (cm^2^)	1.77 (0.95, 3.14)

Data are expressed as median with interquartile range or proportion of patients (%).

Hypokalemia is defined as spontaneous serum potassium concentration <3.5 mmol/L. Abbreviations: SBP, systolic blood pressure; DBP, diastolic blood pressure; BMI, body mass index; ARR, aldosterone to renin ratio.

### Immunohistopathological Subtyping of Unilateral PA Using CYP11B2 Immunohistochemistry

**FIGURE 1:**
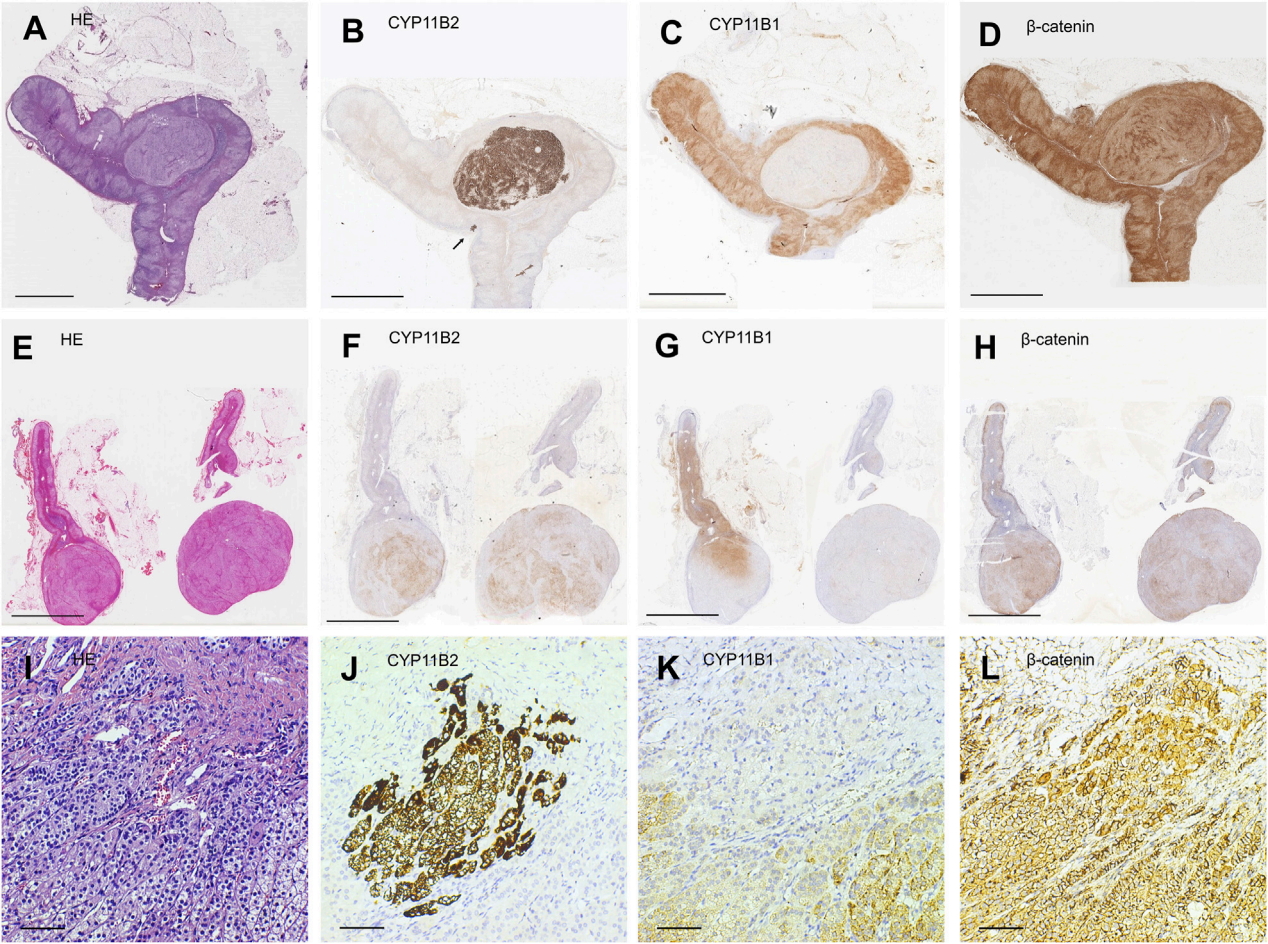
Representative illustration of different subtypes of unilateral primary aldosteronism. **(A,E,I)** Hematoxylin-eosin staining. **(B,F,J)** Immunostaining for CYP11B2. **(C,G,K)** Immunostaining for CYP11B1. **(D,H,L)** Immunostaining for β-catenin. Scale bars, 5 mm **(A**–**H)** and 50 μm **(I**–**L)**. **(A**–**D)** Representative illustration of aldosterone-producing adenoma (APA). **(E**–**H)** Representative illustration of multiple APA. **(I**–**L)** Representative illustration of an aldosterone-producing cell cluster (APCC) whose site is indicated by a black arrow in **(B)**.

HE staining revealed that 77.6% (104/134) of the patients were diagnosed with adenoma and 22.4% (30/104) with unilateral adrenal hyperplasia (UAH). Among 104 patients with adenoma, 91 showed positive CYP11B2 immunostaining in their tumors; whereas 11 patients showing multiple CYP11B2-positive immunostaining tumors were considered as multiple APAs (Figure 1). One patient showed APCC in the ZG with CYP11B2-negative unilateral adrenocortical adenomas. The remaining one patient showed neither positive CYP11B2 immunostaining adenoma nor APCC on his section (Supplementary Figure S1). Among 30 patients with UAH, 27 patients showed one positive CYP11B2 staining nodule; consequently, their diagnosis was changed to APA. Three patients showed APCC in their adrenal cortex without CYP11B2-positive adrenocortical nodules or diffuse CYP11B2-positive ZG hyperplasia.

Therefore, the final diagnosis of these 134 patients with unilateral PA was classified into four subtypes according to CYP11B2 immunostaining: 1).118 unilateral single APA, 2).11 patients with unilateral multiple APAs, 3). four patients with APCC, and 4). one patient with unilateral PA of undefined source (Table 2).

**TABLE 2:** Subtype classification based on CYP11B2 immunostaining result of unilateral primary aldosteronism.

Hematoxylin-eosin staining	n	CYP11B2 immunostaining result	Subtype classification
adenoma	91	Single CYP11B2-positive adenoma	Single APA
adenoma	11	Multiple CYP11B2-positive adenomas	Multiple APAs
adenoma	1	CYP11B2 negative in adenoma, APCC in cortex	APCC
adenoma	1	CYP11B2 negative in whole section	Untargetable culprit
UAH	27	Single CYP11B2-positive nodule	Single APA
UAH	3	CYP11B2 negative in nodular hyperplasia, APCC in cortex	APCC

UAH, unilateral adrenal hyperplasia; n, number of patients; APCC, aldosterone-producing cell cluster; APA, aldosterone-producing adenoma.

### CYP11B2/1 Immunostaining Score and Correlation Between CYP11B2/1 Staining Score and Clinical Characteristics

In the semiquantitative analysis of CYP11B2 immunostaining, illustration of various intensities of CYP11B2 staining in unilateral APA specimens is demonstrated in Supplementary Figure S2. Tumor area was calculated according to the diameter of the adrenocortical tumor on CT with the assumption that the tumor was approximately spherical in shape. As both tumor area and steroidogenic enzyme immunoreactivity are determinants of the overall tumor steroid hormone production, we investigated the functional significance of CYP11B2 by multiplying the H-score of the steroidogenic enzyme by tumor area and correlated the product with clinical parameters of the 118 unilateral single APA patients. The H-score of CYP11B2 adjusted for tumor area was positively correlated with serum aldosterone (r = 0.399, *p* = 0.000) and ARR (r = 0.301, *p* = 0.001), and inversely correlated with serum potassium (r = −0.236, *p* = 0.010). The CYP11B2 immunoreactivity adjusted for tumor area was not correlated with serum cortisol (r = 0.158, *p* = 0.097) or PRA (r = −0.168, *p* = 0.073; Table 3).

**TABLE 3:** Results of Spearman’s correlation test between immunohistochemical staining scores and clinical parameters.

		Aldosterone	PRA	ARR	Potassium	Cortisol
Adjusted CYP11B2 score	r	0.399	−0.168	0.301	−0.236	0.158
	*P*	0.000	0.073	0.001	0.010	0.097
Adjusted CYP11B1 score	r	0.308	−0.201	0.302	−0.079	0.434
	*P*	0.001	0.031	0.001	0.398	0.000

Abbreviations: r, Spearman correlation coefficient; PRA, plasma renin activity; ARR, aldosterone to renin ratio.

On the other hand, the correlation between CYP11B1 staining and clinical parameters revealed that the adjusted CYP11B1 H-score was positively correlated with serum cortisol (r = 0.434, *p* = 0.000), serum aldosterone (r = 0.308, *p* = 0.001), and ARR (r = 0.302, *p* = 0.001), and inversely correlated with PRA (r = −0.201, *p* = 0.031). The CYP11B1 immunoscore adjusted for tumor area was not correlated with serum potassium (r = −0.079, *p* = 0.398; Table 3).

### β-catenin Immunostaining and the Difference in Clinical Characteristics Between Wild-type and Abnormal β-catenin Staining Groups

**FIGURE 2:**
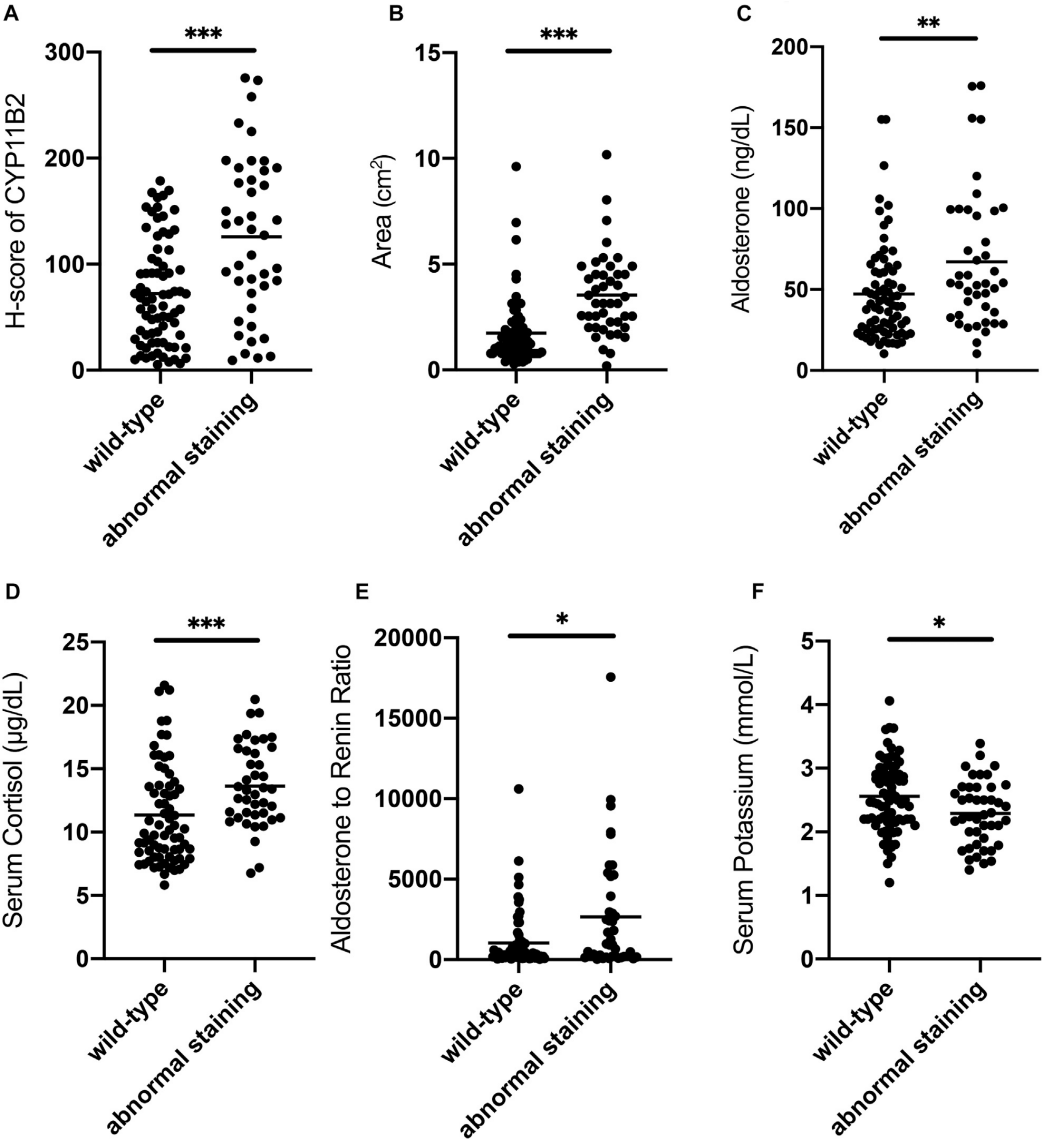
Comparison of clinical characteristics between wild-type staining and abnormal staining groups of β-catenin in patients. **(A)** CYP11B2 H-score of the abnormal staining group is significantly higher than that of the wild-type staining group. **(B)** Tumor area of the abnormal staining group is significantly higher than that of the wild-type staining group. **(C)** Serum aldosterone of the abnormal staining group is significantly higher than that of the wild-type staining group. **(D)** Serum cortisol of the abnormal staining group is significantly higher than that of the wild-type staining group. **(E)** ARR of the abnormal staining group is significantly higher than that of the wild-type staining group. **(F)** Serum potassium of the abnormal staining group is significantly lower than that of the wild-type staining group. Bars in the figure represent the median of each group data. *p < 0.05, **p < 0.01, ***p < 0.001.

Wild-type staining of β-catenin was observed in 64.4% (76/118) of patients with APA and the remaining 35.6% (42/118) showed abnormal β-catenin staining (Supplementary Table S1). We further compared the preoperative clinical and endocrinological parameters of the patients between the wild-type staining and abnormal staining groups (Supplementary Table S1). Preoperative serum aldosterone (*p* = 0.004), ARR (*p* = 0.029), serum cortisol (*p* = 0.000), duration of hypertension (*p* = 0.019), tumor diameter (*p* = 0.000), and tumor area (*p* = 0.000) were significantly higher in the abnormal staining group than that in the wild-type group. Serum potassium level (*p* = 0.032) was significantly lower in the abnormal staining group than that in the wild-type staining group. However, there were no significant differences in SBP (*p* = 0.505), DBP (*p* = 0.504), age (*p* = 0.146), sex (*p* = 0.471), family history of hypertension (*p* = 0.763), BMI (*p* = 0.800), and PRA (*p* = 0.208) between these two groups. As for the immunoreactivity score, the CYP11B2 score (*p* = 0.000) was significantly higher in the abnormal staining group than that in the wild-type staining group, whereas the CYP11B1 score was similar (*p* = 0.262) between these two groups (Figure 2).

### Comparison Between and Predictive Factors Associated With Different Clinical Outcome Groups

Ninety-six of 118 APA patients were successfully followed up to assess the outcome after adrenalectomy for unilateral primary aldosteronism and they were categorized into complete and incomplete clinical success groups as described in the Methods section. Clinical characteristics and immunostaining features of the two groups are compared in Table 4. Gender and family history of hypertension differed significantly between the two groups. In the complete clinical success group, the age (*p* < 0.05), BMI (*p* < 0.05), adjusted CYP11B2 H-score (*p* < 0.05) and adjusted CYP11B1 H-score (*p* < 0.05) were significantly lower than those in the incomplete clinical success group. There were no significant differences in the duration of hypertension (*p* = 0.30), SBP (*p* = 0.07), DBP (*p* = 0.25), serum aldosterone (*p* = 0.83), PRA (*p* = 0.49), ARR (*p* = 0.58), serum potassium (*p* = 0.41), serum cortisol (*p* = 0.07), tumor diameter (*p* = 0.21), tumor area (*p* = 0.21) and β-catenin staining status (*p* = 0.64) between the two groups. Unadjusted and adjusted logistic regression analyses were used to identify factors associated with different clinical outcomes (Supplementary Table S2). The unadjusted logistic regression analysis showed that age, sex, BMI and family history of hypertension were associated with complete clinical success, but adjusted CYP11B2 H-score and adjusted CYP11B1 H-score were not. Finally, we identified three independent predictive factors associated with complete clinical success using an adjusted analysis: age, sex and family history of hypertension, while BMI, adjusted CYP11B2 H-score and adjusted CYP11B1 H-score were not independently associated with complete clinical success (Supplementary Table S2).

**TABLE 4:** Comparison of clinical characteristics and immunohistochemical parameters between different clinical outcome groups in APA patients.

	Complete clinical success (n = 51)	Incomplete clinical success (n = 45)	*P*
Age (years)	46 (35, 54)	51 (42, 56)	<0.05
Male (%)	33.3	60.0	<0.01
Duration of hypertension (months)	84 (24, 120)	96 (48, 132)	0.30
Family history of hypertension (%)	39.2	77.8	<0.001
SBP (mmHg)	160 (150, 180)	173 (150, 195)	0.07
DBP (mmHg)	100 (90, 110)	100 (100, 115)	0.25
BMI (kg/m^2^)	22.86 (20.20, 24.89)	23.44 (22.23, 26.64)	<0.05
Aldosterone (ng/dl)	43.02 (28.63, 73.91)	50.37 (26.16, 71.13)	0.83
Renin activity (ng/ml·h)	0.08 (0.03, 0.21)	0.11 (0.03, 0.28)	0.49
ARR	659.27 (169.90, 2,358.00)	328.24 (153.74, 2,283.51)	0.58
Serum potassium (mmol/L)	2.40 (2.10, 2.70)	2.50 (2.10, 2.96)	0.41
Prevalence of Hypokalemia (%)	98.0	95.6	0.16
Serum cortisol (µg/dl)	10.97 (8.06, 13.69)	12.24 (10.42, 16.12)	0.07
Tumor diameter (cm)	1.50 (1.00, 2.00)	1.60 (1.25, 2.22)	0.21
Tumor area (cm^2^)	1.77 (0.79, 3.14)	2.01 (1.23, 3.87)	0.21
Adjusted CYP11B2 score	95.18 (39.16, 251.11)	186.40 (80.58, 415.28)	<0.05
Adjusted CYP11B1 score	128.59 (58.56, 232.48)	173.22 (113.70, 329.29)	<0.05
β-catenin wild-type staining (%)	64.7	60.0	0.64

Data are expressed as median with interquartile range or proportion of patients (%).

Hypokalemia is defined as spontaneous serum potassium concentration <3.5 mmol/L. Abbreviations: SBP, systolic blood pressure; DBP, diastolic blood pressure; BMI, body mass index; ARR, aldosterone to renin ratio.

## Discussion

Herein, our study firstly evaluated the association between steroidogenic enzyme immunoreactivity, β-catenin immunostaining status, clinical parameters and outcome in patients with unilateral PA. Differential diagnosis between unilateral APA and UAH can sometimes be puzzling and difficult when using the traditional HE staining technique, because the ZG of the peritumoral tissue adjacent to APA can sometimes display different levels of hyperplasia, and HE staining cannot identify the precise lesion that is responsible for aldosterone overproduction (Ganguly, 1998; Enberg et al., 2004; Boulkroun et al., 2010; Nakamura et al., 2014; Yamazaki et al., 2017). *In situ* hybridization of CYP11B2 has been used to identify aldosterone-producing cells; however, this technique cannot be performed at a large-scale as it is time-consuming and difficult to implement. Using a specific monoclonal antibody for CYP11B2, immunohistochemistry staining of steroidogenic enzyme can be an advantageous tool to precisely localize the cells producing aldosterone and markedly improve the diagnostic accuracy as it is faster, more convenient and less expensive than *in situ* hybridization (Nanba et al., 2013; Gomez-Sanchez et al., 2014). Here, 30 patients were initially diagnosed with UAH based on HE staining, and then 27 of them were re-diagnosed with APA after CYP11B2 immunostaining, as positive nodular staining was observed. Moreover, we found more than one APAs on the tissue sections of 11 patients via CYP11B2 immunostaining, many of which were undetectable under CT examination. Yamazaki et al. (Yamazaki et al., 2017) classified CT-negative PA into two subtypes based on CYP11B2 immunostaining: multiple adrenocortical micronodules and diffuse hyperplasia. They also reported that the ZG of adjacent adrenals near micronodules often demonstrated various degrees of paradoxical hyperplasia, which is histopathologically considered hyperplasia but is negative for CYP11B2 staining. Although all patients in our study showed positive CT findings, our result is consistent with that of Yamazaki et al. Paradoxical hyperplasia of ZG cells was also observed in our study. Interestingly, neither diffuse CYP11B2-positive hyperplasia of ZG cells nor positive nodular staining was observed in the remaining three UAH patients; but several APCCs within the adrenal cortex were observed on their sections. Nishimoto et al. (Nishimoto et al., 2010) firstly identified a novel cell cluster positive for CYP11B2 immunostaining, which they termed as APCC. Several studies (Nishimoto et al., 2010; Nanba et al., 2017; Omata et al., 2017; Omata et al., 2018) have demonstrated that APCCs frequently harbor aldosterone-driver somatic mutations mostly in the *CACNA1D* gene, and can autonomously secrete mineralocorticoid independent of the renin-angiotensin system under normal and pathological conditions. Omata et al. (Omata et al., 2018) elucidated that idiopathic hyperaldosteronism (IHA) may result from the accumulation and enlargement of APCCs instead of hyperplasia of ZG cells. Moreover, Nanba et al. (Nanba et al., 2013) observed APCCs in unilateral PA patients with ipsilateral adrenocortical tumor negative for CYP11B2 staining. According to these findings, we hypothesized that APCCs may be the source of aldosterone excess in certain patients with unilateral PA rather than diffuse hyperplasia of aldosterone-producing ZG cells or adenomas. Additionally, one patient in our study had a CYP11B2-negative adrenocortical tumor and one APCC on his section, indicating that APCC rather than the adrenocortical tumor found by CT may be responsible for the aldosterone overproduction. Thus, our findings, in accordance with other studies, indicated that CYP11B2 staining can more precisely pinpoint the culprit responsible for aldosterone overproduction compared to traditional HE staining and effectively differentiate between different subtypes of unilateral PA. Further, APCC may play a crucial role not only in normal blood pressure maintenance (Nanba et al., 2017; Omata et al., 2017) but also in both unilateral and bilateral, CT-negative and CT-positive hyperaldosteronism (Nanba et al., 2013; Yamazaki et al., 2017; Omata et al., 2018).

Immunoreactivity was assessed using the McCarty H-score, which is now considered the best available approach for semi-quantitatively evaluating the immunoreactivity of steroidogenic enzymes in an objective manner in a specific tumor sample (McCarty et al., 1985). In our study, the CYP11B2 H-score correlated with serum aldosterone and serum potassium, but not with PRA, ARR, or serum cortisol. Meanwhile, the CYP11B1 H-score correlated with serum cortisol, but not with serum aldosterone, PRA, or ARR (data not shown). Several studies (Nanba et al., 2013; Nakamura et al., 2014; Ono et al., 2014) have shown that steroidogenic enzyme H-score level alone does not necessarily represent the overall steroid hormone production capacity of an adrenocortical tumor because it only represents the hormone production ability of the tumor per unit area and per cell, and there may be several other factors such as tumor area and upstream steroidogenic enzymes of CYP11B2/1 that may also play a role in hormone production. Therefore, the functional significance of CYP11B2/1 immunostaining was investigated by correlating the H-score adjusted for tumor area with the endocrine data. Through this adjustion, the adjusted CYP11B2 score was correlated with serum aldosterone, ARR, and serum potassium, and the adjusted CYP11B1 score was correlated with serum 8:00 am cortisol, serum aldosterone, ARR, and PRA. These correlationships provide further evidence that adjusted H-score can be used as a new marker to reflect clinical manifestations and severity of unilateral PA.

APA is a heterogenous lesion composed of cells originating from different adrenocortical zones. Both ZF-like cells (cells with large, vacuolated, lipid-laden cytoplasm and central, round nuclei similar to that of ZF) and ZG-like cells (cells with relatively small, lipid-poor cytoplasm and a higher nuclear to cytoplasmic ratio similar to that of ZG) are observed in the majority of APAs (Enberg et al., 2004; Nakamura et al., 2014; Gioco et al., 2015; Fallo et al., 2017). Our results were consistent with the histopathological findings and provide further immunohistochemical evidence on this perspective. Functionally, CYP11B2 is restricted in ZG, catalysing the final steps of mineralocortical biosynthesis, and CYP11B1 is restricted in ZF and zona reticularis (ZR), catalysing the final step of glucocorticoid formation (Stowasser and Gordon, 2016). Our results suggest that APA can simultaneously produce cortisol in patients with PA, which may lead to clinical or subclinical manifestation of Cushing syndrome (Goupil et al., 2015; Stowasser and Gordon, 2016; Fallo et al., 2017). A positive correlationship between the adjusted CYP11B1 score and serum cortisol was revealed in our study, which emphasized the importance of careful monitoring of the hypothalamic-pituitary-adrenal axis before and after surgery in patients with unilateral PA.

Interestingly, the adjusted CYP11B1 score not only correlated with serum cortisol, but also with serum aldosterone, ARR, and PRA. This correlation raised a question on the role of the CYP11B1 enzyme in the biosynthesis of aldosterone. We suggest that both CYP11B1 and CYP11B2 can convert deoxycorticosterone to corticosterone via 11β-hydroxylase, and corticosterone is the precursor of aldosterone. Although CYP11B2 is the only enzyme that can convert corticosterone to aldosterone, CYP11B1 may still contribute to aldosterone overproduction via synthesizing enough precursor, i.e. corticosterone, as a substrate for the biological activity of CYP11B2 (Nakamura et al., 2014; Ono et al., 2014).

The Wnt/β-catenin signaling pathway plays a pivotal role in the regulation of proliferation, differentiation, and function of the normal adrenal cortex and adrenocortical tumorigenesis (El Wakil and Lalli, 2011; Clevers and Nusse, 2012). Aberrant cytoplasmic and nuclear accumulation of β-catenin reflects the constitutive activation of this pathway (Tissier et al., 2005). The prevalence of abnormal β-catenin staining in our study was 35.6%, similar to that reported by Tissier et al. (Tissier et al., 2005). Further, we observed that the abnormal staining group secreted more aldosterone and cortisol than the wild-type staining group. Our results showed that the tumor area and CYP11B2 H-score were significantly higher in the abnormal staining group than those in the wild-type group. Several factors such as McCarty H-score and tumor area are involved in the overall steroid hormone generation of adrenocortical adenoma as described above. General steroid hormone production of APA simultaneously increases as tumor area becomes larger. Besides, higher CYP11B2 H-score implies that constitutive β-catenin activation increases the aldosterone synthesis capacity of APA per unit area and per cell. Studies have revealed that β-catenin can modulate the membrane potential and intracellular ion homoeostasis in different cell types through interacting with ion channels and transporters (Lesage et al., 2004; Wilmes et al., 2012; Zhao et al., 2014). Hence, we hypothesized that an aberrant cytoplasmic accumulation of β-catenin may depolarize the membrane potential and increase the cytosolic Ca^2+^ concentration of APA cells, thereby enhancing the steroidogenic ability. However, this hypothesis needs deeper exploration. Therefore, both the higher CYP11B2 H-score and tumor area effect synergistically give rise to higher serum aldosterone concentration in the abnormal staining group, whereas the higher serum cortisol in the abnormal staining group could mainly result from the tumor area effect rather than the similar CYP11B1 H-score compared with the wild-type group. Nevertheless, the underlying mechanism of why aberrant activation of the Wnt/β-catenin signaling pathway selectively enhances CYP11B2 expression rather than CYP11B1 in patients with APA warrants further investigation.

Laparoscopic adrenalectomy is now considered the standard approach in the treatment of unilateral PA as it is minimally invasive and can normalize aldosterone secretion, decrease blood pressure, and reduce antihypertensive medication dosage (Funder et al., 2008; Stowasser and Gordon, 2016). However, normotension without antihypertensive drug usage cannot always be achieved after adrenalectomy for unilateral PA. Complete clinical success was achieved in 37% of patients in the PASO study (ranging from 17 to 62% between different centers) (Williams et al., 2017). In the current study, complete clinical success was attained in 53.1% patients, similar with the PASO study. Laparoscopic adrenalectomy can confer a decrease in SBP about 25–40 mmHg and a reduction in the number of antihypertensive medications prescribed about 1–2 drug classes averagely (Steichen et al., 2012; Williams et al., 2017). Clinical characteristics predicting hypertension cure include sex, age, family history of hypertension, hypertension duration, BMI, estimated glomerular filtration rate, evidence of arteriolosclerosis and so on (Steichen et al., 2012; Stowasser and Gordon, 2016; Williams et al., 2017). Using the multivariate logistic regression model, our results proposed that sex, age and family history of hypertension were independently associated with clinical outcome. Patients who were younger, female and without a family history of hypertension had a higher likelihood of being cured of hypertension after adrenalectomy. Although the adjusted CYP11B2 and CYP11B1 H-scores were not independent predictors associated with clinical success based on multivariate analysis in our study, our results demonstrated that the adjusted H-scores in the complete success group were significantly lower than those in the incomplete success group, indicating that patients with lower immunostaining scores might benefit more from surgery for unilateral PA. It is reported that hyperaldosteronism patients have an increased rate of cardiovascular events than matched essential hypertension patients, showing that aldosterone per se can cause target organ damage independent from its hypertensive effect (Mulatero et al., 2013). The pathogenetic role of aldosterone excess include chronic inflammation, fibrosis, endothelial dysfunction, glucose and lipid metabolism disorder (Kozakova et al., 2003; Fallo et al., 2007; Staermose et al., 2009; Tsuchiya et al., 2009). Through these machanisms, aldosterone oversecretion can result in irreversible vascular and renal damage. Several studies have demonstrated media-to-lumen ratio of small arteries and estimated glomerular filtration rate are independently associated with the outcome following adrenalectomy for unilateral PA (Rossi et al., 2008; Group et al., 2009). As mentioned above, the adjusted H-scores are positively correlated with serum aldosterone. We postulate that patients with higher scores may develop more deleterious vascular remodeling and more impaired renal function due to the persistent secondary hypertension and thereby decrease the likelihood of complete clinical success (Rossi et al., 2008; Group et al., 2009). The main limitaion of our study is that the sample size is not large enough, which maybe undermines the statistical validity of multivariate analysis. Larger studies are needed to identify the association between immunohistochemical features and clinical outcome after adrenalectomy for unilateral PA.

In conclusion, CYP11B2 immunohistochemical staining is a useful histopathological tool for precisely locating the cells responsible for aldosterone production, and assists in the differential diagnosis and subtyping of unilateral PA. In a small portion of patients with unilateral PA, increased autonomous aldosterone production is not caused by the CT-detectable adenomas but might be driven by subcapsular APCCs. Adjusted CYP11B2 and CYP11B1 H-scores are correlated with serum aldosterone and other endocrine parameters in patients with APA. The aberrant constitutive activation of β-catenin can provoke the proliferation activity and enhance the steroidogenic ability of tumor cells, which suggests that a Wnt pathway inhibitor may be a potential, actionable therapeutic option for the treatment of hyperaldosteronism. Being younger, female and without a family history of hypertension predicts a better outcome after adrenalectomy in primary aldosteronism patients. However, further studies are required to elucidate the prognostic significance of immunostaining on surgical outcome of unilateral PA.

## Data Availability

The raw data supporting the conclusion of this article will be made available by the authors, without undue reservation.
