# Supplementary material for: Immunohistochemical Analysis of CYP11B2, CYP11B1 and β-catenin Helps Subtyping and Relates With Clinical Characteristics of Unilateral Primary Aldosteronism
Source: Front Mol Biosci. 2021 Sep 24;8:751770. doi: 10.3389/fmolb.2021.751770 (PMC8497787; doi:10.3389/fmolb.2021.751770)
Supplement: Supplementary file 1 [file DataSheet1.pdf]

Supplementary Material

1 Patients and Methods

1.1 Patients

Adrenal computed tomography (CT) and adrenal venous sampling were performed in patients with PA to differentiate between bilateral and unilateral PA. Cannulation was considered successful if $Cortisol_{adrenal\ vein}/Cortisol_{peripheral\ vein}$ was >3 without adrenocorticotropic hormone (ACTH) stimulation. Cortisol-corrected aldosterone (A/C) ratio was used to correct adrenal venous aldosterone levels for differing degrees of dilution of adrenal vs. peripheral venous blood. Lateralization was defined as the ratio of (A/C) adrenal vein vs. (A/C) contralateral adrenal vein >2 without ACTH stimulation. Patients with unilateral aldosterone overproduction underwent laparoscopic adrenalectomy.

1.2 Immunohistochemistry

McCarty H-score was used to evaluate the immunoreactivity semi-quantitatively in our study. Firstly, we assessed 500 parenchymal cells in each region of each case, and the percentage of immunopositive cells was calculated. Secondly, the immunointensity of the stained cells for each specific steroidogenic enzyme was relatively classified into four grades as: not detectable (0), weak but detectable above control (1+), distinct (2+), and very strong (3+). The percentage of stained cells multiplied by the corresponding immunointensity equaled the McCarty H-score. Further,in all APA sections examined, three independent observers (Y.J., L.S., and J.X.) assessed the H-scores and obtained the averages in the blind approach. And all three observers were blinded to the clinical data of each patient. The immunostained slides were simultaneously reassessed using a multiheaded microscope in case of a discordance until a consensus was reached.

2 Supplementary Figures

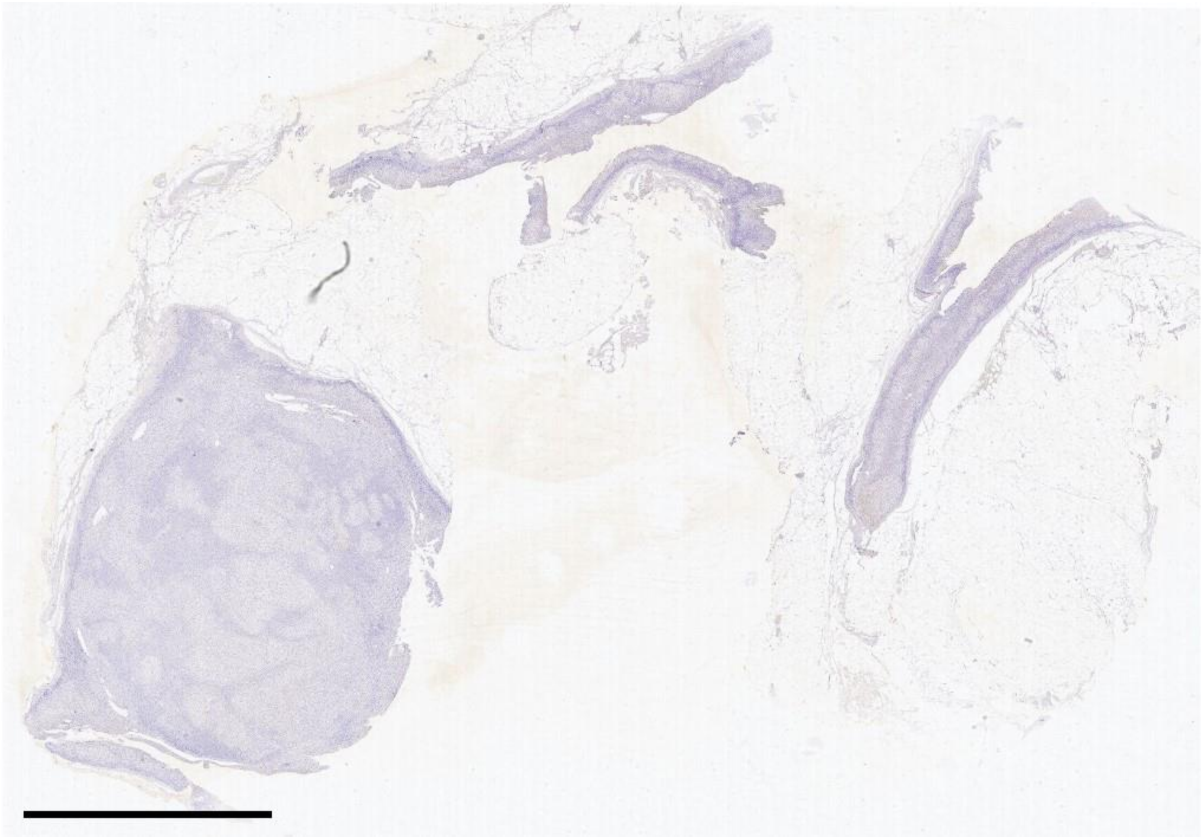

Supplementary Figure 1. CYP11B2 immunohistochemical image of tissue section of the patient with untargetable culprit. Scale bar, 5 mm.

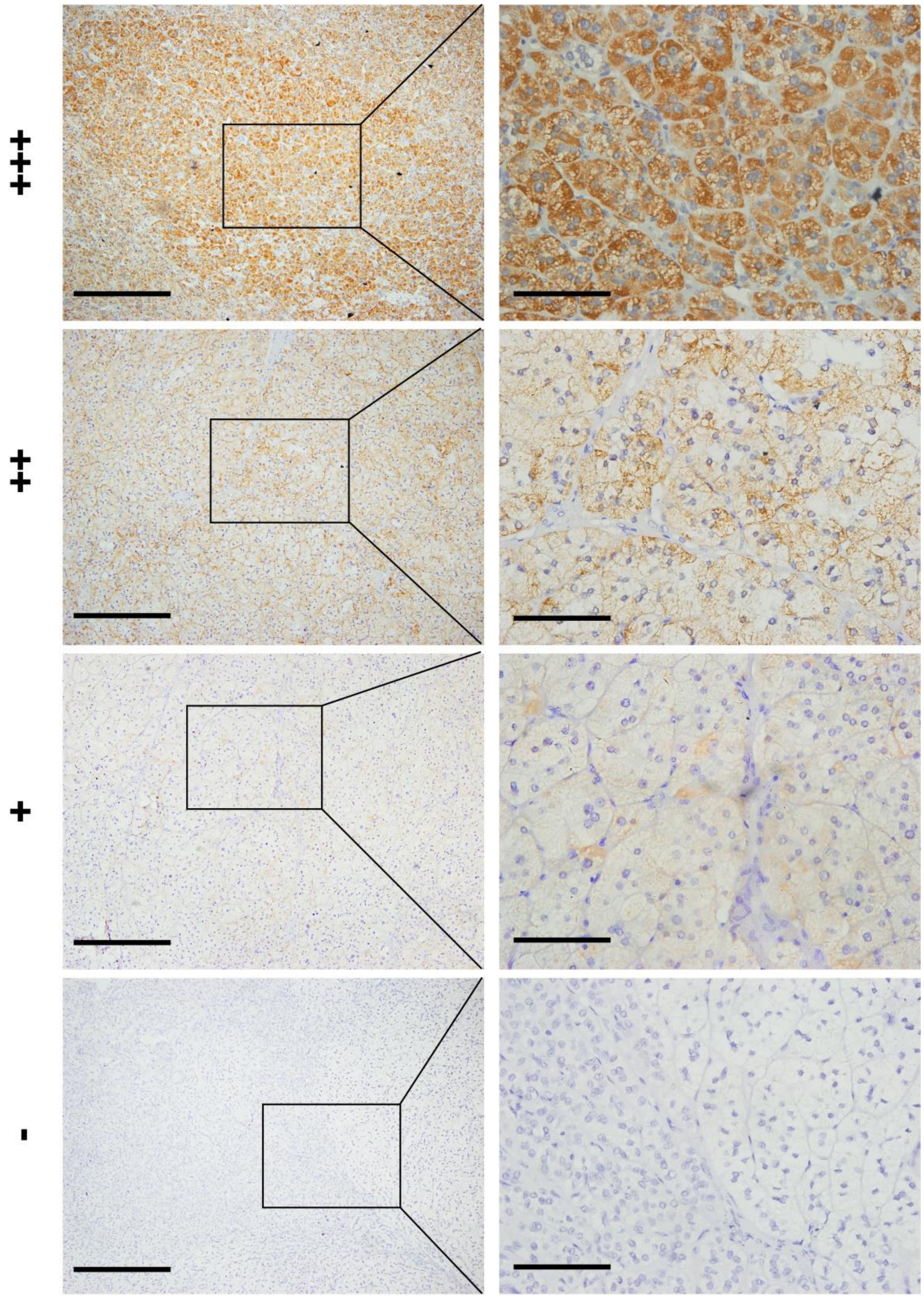

Supplementary Figure 2. Illustration of different immunointensities of CYP11B2 immunostaining. Scale bars, 200 μm (left column) and 50 μm (right column). Magnification 100× (left column) and 400× (right column).

3 Supplementary Tables

Supplementary Table 1. Comparison of clinical characteristics between the wild-type staining and abnormal staining groups of β-catenin

	Wild-type staining group (n = 76)	Abnormal staining group (n = 42)	*P*
Age (years)	46 (36, 55)	51 (44, 55)	0.15
Male (%)	47.4	40.5	0.47
Duration of hypertension (months)	60 (27, 120)	120 (45, 180)	<0.05
Family history of hypertension (%)	55.3	52.4	0.76
SBP (mmHg)	160 (150, 180)	172 (150, 180)	0.51
DBP (mmHg)	100 (100, 110)	100 (90, 110)	0.50
BMI (kg/m^2)	23.21 (21.22, 25.38)	23.00 (21.15, 25.64)	0.80
Aldosterone (ng/dL)	39.87 (24.27, 60.93)	53.86 (33.83, 98.71)	<0.01

Renin activity (ng/mL·h)	0.12 (0.05, 0.21)	0.07 (0.02, 0.24)	0.21
ARR	361.19 (168.67, 1012.03)	946.95 (196.45, 4251.62)	<0.05
Serum potassium (mmol/L)	2.50 (2.19, 2.90)	2.26 (1.88, 2.70)	<0.05
Prevalence of Hypokalemia (%)	96.1	100.0	0.91
Serum cortisol (μg/dL)	10.05 (8.05, 13.63)	12.96 (11.14, 16.60)	<0.001
Tumor diameter (cm)	1.34 (1.00, 1.66)	2.00 (1.60, 2.40)	<0.001
Tumor area (cm^2)	1.40 (0.79, 2.17)	3.14 (2.01, 4.52)	<0.001
CYP11B2 H-score	66.09 (26.94, 104.70)	130.06 (69.09, 188.92)	<0.001
CYP11B1 H-score	83.10 (57.15, 96.91)	83.39 (66.60, 122.03)	0.26

Data are expressed as median with interquartile range or proportion of patients (%). Hypokalemia is defined as spontaneous serum potassium concentration <3.5 mmol/L. Abbreviations: SBP, systolic blood pressure; DBP, diastolic blood pressure; BMI, body mass index; ARR, aldosterone to renin ratio.

Supplementary Table 2. Factors associated with clinical outcomes after adrenalectomy for unilateral primary aldosteronism

	Complete clinical success versus Incomplete clinical success			
	Unadjusted model		Adjusted model	
	Odds ratio (95% CI)	***P***	Odds ratio (95% CI)	***P***
Age	0.96 (0.92- 0.99)	<0.05	0.95 (0.91- 0.99)	<0.05
Gender	0.33 (0.15- 0.77)	<0.05	0.31 (0.13- 0.73)	<0.01
BMI	0.85 (0.74- 0.97)	<0.05	0.90 (0.79- 1.03)	0.13
Family history of hypertension	0.18 (0.08- 0.45)	<0.001	0.25 (0.10- 0.64)	<0.01
Adjusted CYP11B2 score	1.00 (0.99- 1.00)	0.12	0.99 (0.99- 1.00)	0.18
Adjusted CYP11B1 score	1.00 (0.99- 1.00)	0.18	0.99 (0.99- 1.00)	0.37
